# Intimate relationship satisfaction among Swedish police officers: work centrality, workplace social climate, and the conditional role of external support

**DOI:** 10.3389/fpsyg.2026.1778017

**Published:** 2026-07-09

**Authors:** Arian Rostami, Jonas Hansson

**Affiliations:** Umeå University, Unit of Police Work, Umeå, Sweden

**Keywords:** intimate relationship satisfaction, police officer, social support, work centrality, work social climate

## Abstract

Policing is a high-demand profession that can intensify work–family conflict, significantly affecting satisfaction in intimate relationships. This cross-sectional study investigated intimate relationship satisfaction among Swedish police officers and examined the influence of psychosocial and organizational factors. Data were collected from police officers using the ENRICH Marital Satisfaction (EMS) scale and the QPS Nordic questionnaire. Results indicated a moderate level of intimate relationship satisfaction among police officers, independent of sociodemographic variables. A positive social climate and support from friends and relatives were positively associated with relationship satisfaction, while work centrality was most strongly and negatively associated with relationship satisfaction. A significant interaction between work centrality and support from friends and relatives showed that the benefit of external support was strongest when work centrality was high, indicating its conditional and compensatory role. Police organizations should adopt a holistic strategy to manage the impact of work centrality, integrating policies that strengthen positive work social climate and officers’ external support networks to foster sustainable work–family balance in policing.

## 1 Introduction

Satisfaction with intimate relationships, such as marriage or cohabiting relationships, is one of the most important predictors of overall life quality and various areas of adult life (Lee and Park, 2023). While personal and interpersonal factors greatly affect one’s satisfaction in an intimate relationship, research increasingly indicates that job characteristics also are important in shaping marital and partner relationship outcomes. Job-related stress can spill over into personal relationships, impacting individual well-being and relationship dynamics (Haines et al., 2006; Randall and Bodenmann, 2017). Conflicts between work and family life are associated with lower marital and relationship satisfaction. This link is influenced by various work-related factors, such as time pressure, limited control at work, lack of support, and other job strains that transfer from the workplace to home life (Grosswald, 2004; Fallahchai et al., 2019). These effects can be stronger in high-demand jobs like police work, where irregular shifts, high occupational stress, and heavy workloads often intensify work–family conflict and add more strain to relationships (Granholm Valmari et al., 2023).

These occupational stressors do not remain limited to the workplace; they often spill over into personal and family life (Mikkelsen and Burke, 2004; Miller, 2021; Watson et al., 2025), influencing the quality of intimate relationships and overall marital satisfaction (Roberts et al., 2013; Tuttle et al., 2018). Research indicates that police officers face substantial challenges in balancing their professional duties and family responsibilities, including feeling torn between work and family roles, grappling with the overwhelming demands of their profession, maintaining constant alertness and vigilance, and managing guilt resulting from conflicts between work and family life (Granholm Valmari et al., 2023).

Police officers have to deal with a distinctive combination of operational and organizational stressors along with societal responsibilities and strict legal regulations (Violanti and Paton, 1999; Gomes and Afonso, 2016; Violanti et al., 2017). Their work is characterized by irregular shift schedules (Charles et al., 2007; Ma et al., 2015), frequent exposure to acute and cumulative trauma (Acquadro Maran et al., 2020; Papazoglou et al., 2022), and a persistent need for emotional regulation in high-pressure environments (Nero et al., 2022; Hansson and Padyab, 2023; Sunday, 2024). Organizational stressors within police departments—such as inadequate resources, unsupportive supervision, bureaucratic constraints, and workplace culture issues—often produce more sustained strain in police officers’ everyday work life (Shane, 2010; Houdmont, 2017).

In recent years, Sweden has experienced a sharp increase in violent incidents among criminals (Rostami and Sarnecki, 2022), including public shootings (Ceccato et al., 2024) and residential explosions (Sturup et al., 2020), placing unprecedented pressure on law enforcement. Furthermore, the Swedish police have undergone a major centralization reform in recent years, aiming to create a unified national authority in response to the fragmentation and inefficiencies of the previous decentralized system. This change has also resulted in notable challenges in the workplace, such as high workloads and an increasing lack of resources and support (Statskontoret, 2018). These dynamics create a complex environment in which officers must navigate increasing work demands along with personal and relational stressors. This context helps understand the heightened organizational pressure Swedish police officers are experiencing and sheds light on how factors in the work environment may affect their family life. Despite the critical role of work-related factors for intimate relationship satisfaction, the link between the two remains underexplored in the context of police work, particularly in Swedish policing. Empirical studies that explore these interdependent domains are crucial, both for developing a scientific understanding of work–family dynamics and for informing practical improvements within police organizations that may support police officers’ personal lives.

Accordingly, this study aims to address this gap by examining intimate relationship satisfaction among a group of Swedish police officers and how psychological, social, and organizational factors in police work are associated with officers’ individual satisfaction with their intimate relationships.

We utilized the umbrella term “intimate relationship satisfaction” throughout this study to reflect the diverse relationship statuses within the police population, aligning with modern family relationships. Although the data were collected using an instrument rooted in marriage research (the ENRICH Marital Satisfaction scale), the conceptual focus remained on the quality of the most committed intimate relationship (married and cohabited relationships). This comprehensive approach enhances the ecological validity of the findings, ensuring their relevance to the variation of committed relationships held by police officers in Sweden.

### 1.1 Theoretical framework

To understand the interplay between work and intimate partner relationships, we apply two theoretical frameworks: work–family conflict theory and the conservation of resources theory. As conceptual lenses, these frameworks allow us to explore how specific psychosocial and organizational work characteristics can predict intimate relationship satisfaction as a family life outcome.

Work–family conflict theory provides a robust theoretical framework for understanding the relationship between the police work environment’s characteristics and police officers’ intimate relationships. This theory explains that work–family conflict occurs when the demands of work and family roles are incompatible (Greenhaus and Beutell, 1985). This conflict can flow in two directions: from work to family (work-to-family conflict) and from family to work (family-to-work conflict). According to Byron (2005), work-related factors usually trigger work-to-family conflict, whereas non-work factors are more likely to lie behind family-to-work conflict. According to the work–family conflict theory, there are three types of conflict that transfer from work to family life through various psychological and behavioral pathways: time-based, strain-based, and behavior-based conflict. Time-based conflict arises when the time devoted to work limits the ability to meet family responsibilities, either due to long hours at the office or because of mental preoccupation with work while at home. Strain-based conflict occurs when stress and negative emotions from work spill over into family life and adversely affect relationships. Behavior-based conflict is when behaviors that are effective or rewarded at work cannot meet the requirements of a family role or even are incompatible with them, leading to conflict and strained relationships (Greenhaus and Beutell, 1985).

Complementing this, the conservation of resources theory (Hobfoll, 1989) explains stress as a process of resource loss and gain. Individuals strive to obtain, protect, and build valued resources, such as personal characteristics, energy, time, and social relationships. In the context of work and family relationships, multiple roles at work and in the family can deplete resources in fulfilling work and family responsibilities, leading to stress, more conflicts, and dissatisfaction. Also, investing a substantial portion of one’s resources in one domain can deplete the resources available for other domains and increase stress and psychological strains (Grandey and Cropanzano, 1999). According to this theory, although the loss of resources is stressful, individuals may use other resources to make up for the loss (Hobfoll, 1989). Individuals can mitigate work–family conflict through various resource management strategies to navigate the challenges imposed by the demands and reduce the psychological strain associated with work–family conflict. These strategies include establishing clear boundaries between work and family life, managing time effectively, and getting support from inside and outside of work (such as from friends and family members) (Wang, 2024). Therefore, having adequate resources and replenishing one’s resources can buffer job demands, promote positive emotional states, and effectively mitigate work–family conflict and prevent its negative consequences.

Police work is characterized by constant exposure to danger, organizational constraints, irregular schedules, and emotionally taxing incidents. According to the conservation of resources theory (Hobfoll, 1989), such job demands threaten and consume police officers’ valuable resources, leading to stress and emotional exhaustion. In addition to work-related demands, inter-role conflict between the policing role and the family role can further exacerbate the resource loss and intensify stress. Occupational stress does not simply disappear at the end of a shift; instead, officers often carry the emotional and psychological strain from work into their homes and intimate relationships. This spillover increases work–life conflict and reduces intimate relationship satisfaction (Tuttle et al., 2018; Granholm Valmari et al., 2023). As Hobfoll (1989) describes through the concept of loss spirals, police officers use their resources to meet the ongoing job demands, making it harder to manage family responsibilities, which in turn increases work–family conflict. To address this conflict, they need to use additional resources, which intensifies the cycle of resource loss, stress, and work–family conflict over time.

## 2 Methods

This cross-sectional study is based on a project called Mareld, initiated by the Swedish Police Authority. The project aimed to improve the working environment and health of police employees as well as to increase safety and security for authorities, businesses, and citizens living in three police areas in the Stockholm region (Sundqvist et al., 2021). All police employees working in the targeted areas were invited to participate in the study. A set of questionnaires was distributed among the police employees, and a total of 365 of them took part in the first round of the survey (response rate about 75%). Given the differences between police officers and civil servants in the nature of their work, we decided to include only the group of police officers. After excluding 49 civil servants, the sample comprised 316 police officers. As the main focus of the study was on intimate relationship satisfaction, those who were single or in a less committed relationship were excluded. In the end, 252 police officers who were in some form of couple relationship were included in the analysis. Data were collected solely from the officers; no data were obtained from their partners, representing an individual rather than a dyadic assessment of intimate relationship satisfaction.

To ensure participant confidentiality, a rigorous de-identification protocol was followed. Each questionnaire was assigned a unique code number to protect confidentiality. After completion, respondents detached the front page, which included their personal identification number and sociodemographic details, and sent this and the completed questionnaire to the research team in separate sealed envelopes. The research team subsequently matched the data using the unique codes to ensure that identities remained confidential.

The study was approved by the Swedish Ethics Review Authority (No. 2017/516-31). Participation was voluntary, and participants were informed of the aims and procedures of the study and their right to withdraw at any time without consequence. The survey took approximately 60 min to complete. To facilitate participation, questionnaires were completed during regular working hours.

### 2.1 Instruments

Data were collected using a set of questionnaires, including several questionnaires that were relevant to the various aims of the original Mareld project. The data from the work-demographic questions, the ENRICH Marital Satisfaction Inventory, and the General Questionnaire for Psychological and Social Factors at Work (QPS Nordic) were used in this study.

The sociodemographic section of the questionnaire was developed by the research team to collect background information about the respondents. This part included questions on gender, age, marital status, number of children, work experience, type of work, job position, and other sociodemographic and work-related characteristics.

The ENRICH Marital Satisfaction (EMS) scale (Fowers and Olson, 1993), comprising 15 items, is a brief version of the ENRICH Inventory (Fowers and Olson, 1989) designed to assess marital satisfaction. Although the scale was originally designed for married couples, it has been widely used with cohabiting partners and is appropriate for assessing satisfaction in intimate relationships. The applied questionnaire was adapted to include individuals in any committed intimate relationship (married or cohabiting relationships). This adaptation included substituting the term “spouse” with the more inclusive term “partner” in the questionnaire and item wording. The adaptation ensured that the instrument assessed the satisfaction of the most significant intimate relationship in the officer’s life, reflecting the broader scope of contemporary intimate relationships. This shift from “marital” to “intimate relationship” satisfaction reflects the conceptual premise that the functional dimensions of a committed bond remain consistent regardless of formal legal status. In the Swedish cultural context, where cohabitation is a prevalent and stable relationship form, these interpersonal dynamics are considered conceptually equivalent to those found in marriage. Consequently, the EMS was utilized to measure the core dimensions of the diverse forms of committed relationships in this study.

The EMS consists of two subscales: Idealistic Distortion (5 items) and Marital Satisfaction (10 items). The Idealistic Distortion (ID) items reflect the tendency of respondents to answer questions in a socially desirable way and are used to adjust individual scores on marital satisfaction to address this bias. Each of the 10 items measuring Marital Satisfaction (MS) represents one of the dimensions of the marital relationship, including personality issues, equalitarian roles, communication, conflict resolution, financial management, leisure activities, sexual relationship, children and marriage, family and friends, and religious orientation. Respondents answer each item by selecting one option on a Likert scale ranging from 1 (strongly disagree) to 5 (strongly agree). Raw MS and ID scores were converted to percentiles (PMS, PID), following the EMS scoring procedure. To calculate the total EMS score, marital satisfaction was adjusted for idealistic distortion based on the following formula: PMS - [(PMS) * *r*^2^ * (PID * 0.01)] (Fowers and Olson, 1993; Nunes et al., 2022). In the present study, a sample-specific constant was obtained from the correlation between marital satisfaction and idealistic distortion (*r* = 0.82), which represents the shared variance between the two constructs. This sample-specific constant was applied instead of the 0.40 constant reported in the original US-based study (Fowers and Olson, 1993), to reflect the characteristics of the current sample and research context. Higher scores indicate a higher partner relationship satisfaction. In the current sample, Cronbach’s alpha for the 15-item EMS scale was 0.89, indicating internal consistency.

The QPS Nordic has been developed to assess psychological, social, and organizational working conditions (Dallner et al., 1999). The standard questionnaire comprises 129 questions organized as 14 dimensions (including a dimension of personal background) and 26 subscales. The main dimensions measure different working conditions characteristics, including job demands, role expectations, control at work, predictability at work, mastery of work, social interactions, leadership, group work, organizational culture and climate, work centrality, interaction between work and private life, organizational commitment, perception of group work, and work motives. The response options for all questions were based on a Likert scale ranging from 1 to 5; exceptions were the bullying and harassment questions, which had Yes/No response options, and the work centrality questions, which included a Likert scale ranging from 1 to 7 as well as one question requiring respondents to distribute a total of 100 points across five options. Work centrality scores were converted to a 1-to-5 Likert scale during the scoring for consistency in the analysis (Dallner et al., 1999).

In the present study, we used 113 questions (excluding 11 background questions). The dimensions of organizational commitment (3 questions) and interaction between work and private life (2 questions) were not included in the survey instrument. The selection of dimensions was determined during the broader research design phase of the project, in which priority was given to core psychosocial and organizational working conditions. Consequently, questions from these two dimensions were not available for analysis.

### 2.2 Statistical analysis

Descriptive statistics for categorical sociodemographic variables are reported as numbers and percentages. Continuous sociodemographic variables, intimate relationship satisfaction, and subscales of psychological and social factors at work (QPS Nordic) are presented as mean scores and standard deviations. Independent *t*-tests were applied to assess group differences on the continuous variables. Pearson correlation coefficients were used to examine associations between intimate relationship satisfaction and continuous sociodemographic variables as well as psychological and social factors at work. The variables with significant associations with the dependent variable (intimate relationship satisfaction) in bivariate analyses were applied to multiple regression analyses. Raw MS and ID scores were converted to percentiles using SPSS’s Rank Cases with fractional rank as percentage, reflecting the sample’s distribution (*N* = 252). The analysis employed multiple regression to examine the effects of work environment factors on intimate relationship satisfaction. Before the analysis, standard assumptions for multiple regression, including normality of residuals, homoscedasticity, and linearity, were verified through visual inspection of P–P plots and scatterplots of standardized residuals. Multicollinearity was assessed using Variance Inflation Factors (VIF), and predictors were centered to minimize collinearity with the interaction term. Although regression analysis allows for the examination of statistical prediction of the outcome, it does not establish causality. Given the cross-sectional design, the findings should be interpreted as reflecting associations rather than causal relationships.

The analysis was conducted using SPSS Version 29.0.2.0. The moderation analysis was conducted using the PROCESS macro (Version 4.2) for SPSS (Hayes, 2022). The pick-a-point approach was applied to test the significance of the interaction at different levels of the moderator (work centrality), with simple slopes calculated at the 16th, 50th, and 84th percentiles based on the sample mean and standard deviation of work centrality (3.40 ± 1.16) (corresponding to values of 2.00, 3.33, and 4.67).

## 3 Results

### 3.1 Participants’ characteristics and descriptive statistics

To provide context for the findings, this section first presents the sociodemographic characteristics of the participants, followed by the descriptive statistics for the core study variables. As Table 1, presenting the sample characteristics, shows, of the 252 police officers who participated in the study, 68% were male and 32% were female. Their mean age was 37.58 ± 9.35 years, and most of them (76%) held a university degree. Furthermore, 68% of the participants had children. Their work experience was 10.33 ± 9.91 years on average, ranging from less than 1 year to 42 years. Approximately 37% of the respondents held managerial positions, while the majority (63%) worked as patrol police officers and had mixed shift schedules.

**Table 1:** Sociodemographic and work-related characteristics of the participating police officers.

Variables	Mean±SD (Min–Max) or *N* (%)
Gender	
Male	172 (68.3%)
Female	80 (31.7%)
Age	37.58 ± 9.35 (22–65)
Education	
Elementary school & high school	60 (24.4%)
University	187 (75.6%)
Children	
Yes	160 (68.1%)
No	75 (31.9%)
Job experience (years)	10.33 ± 9.91 (0–42)
Managerial position	
Yes	65 (25.9%)
No	186 (74.1%)
Type of work	
External services	156 (63.2%)
Internal services	91 (36.8%)
Shift plan	
Daytime	92 (37.1%)
Two/three-shift work	156 (62.9%)

Descriptive statistics for intimate relationship satisfaction and work environment subscales are presented in Table 2. The mean and standard deviation of the intimate relationship satisfaction score (EMS) were 28.75 ± 13.95. Based on the sample-specific correction factor derived from the shared variance between satisfaction and idealistic distortion (*r^2^* = 0.67), the theoretical midpoint for this study was calculated at 33.25. Analysis of the distribution revealed that the scores were approximately normally distributed, with a skewness of 0.198 and a kurtosis of 0.165. This was further confirmed by a non-significant Kolmogorov–Smirnov test (*p* = 0.097). To provide a descriptive overview of the sample, participants were organized into three categories based on quartile distributions: low intimate relationship satisfaction (bottom 25%: EMS ≤ 19.80, *n* = 64), moderate intimate relationship satisfaction (middle 50%: EMS 19.80–36.97, *n* = 123), and high intimate relationship satisfaction (top 25%: EMS > 36.97, *n* = 61). This quartile-based approach was utilized to ensure the categories were grounded in the specific variance of the population of the current study.

**Table 2:** Means and standard deviations for intimate relationship satisfaction (EMS) and work environment subscales.

Variables	Mean±SD (Min–Max)
Intimate relationship satisfaction	28.75 ± 13.95 (0.39–71.28)
Job demands	
Quantitative demands	3.13 ± 0.72 (1–5)
Decision demands	3.60 ± 0.68 (1.33–5)
Learning demands	2.94 ± 0.59 (1–4.67)
Role expectations	
Role clarity	3.74 ± 0.70 (1.33–5)
Role conflict	3.70 ± 0.73 (1–5)
Control at work	
Positive challenges	4.09 ± 0.62 (2–5)
Control of decisions	2.92 ± 0.63 (1.20–4.6)
Control of work pace	2.84 ± 0.96 (1–5)
Predictability at work	
Predictability next month	2.32 ± 1.07 (1–5)
Predictability 2 years	2.76 ± 1.01 (1–5)
Preference for challenge	3.43 ± 0.72 (1–5)
Perception of mastery	3.98 ± 0.47 (2.5–5)
Social interactions	
Support from supervisor	3.48 ± 0.86 (1–5)
Support from coworkers	4.06 ± 0.69 (2–5)
Support from friends and relatives	3.95 ± 0.82 (2–5)
Bullying	0.79 ± 0.39 (0.50–2.33)
Leadership	
Empowering leadership	3.23 ± 0.97 (1–5)
Fair leadership	4.08 ± 0.79 (1–5)
Organizational culture and climate	
Social climate	3.97 ± 0.72 (1–5)
Innovative climate	3.67 ± 0.69 (1–5)
Inequality	1.96 ± 0.93 (1–5)
Human resources primacy	3.05 ± 0.80(1–5)
Work centrality	3.40 ± 1.16 (0.33–6.67)
Perception of group work	4.11 ± 0.68 (1.67–5)
Work motives	
Intrinsic motivation	3.62 ± 0.56 (1.67–5)
Extrinsic motivation	3.51 ± 0.63 (1.67–5)

Among work environment subscales (Table 2), perceptions of group work, positive challenges at work, support from coworkers, and fair leadership had the highest mean scores. Conversely, inequality and predictability for the next month had the lowest mean scores. The bullying and harassment subscale was not included in this comparison of mean values, as it was measured using a categorical (Yes/No) scale rather than the 5-point Likert scale used for the other subscales.

### 3.2 Bivariate associations with intimate relationship satisfaction

Following the descriptive overview, bivariate analyses were conducted to identify which demographic and work environment factors were correlated with intimate relationship satisfaction. Analyses show no significant associations between intimate relationship satisfaction and any of the sociodemographic variables (Table 3). Female officers reported slightly higher intimate relationship satisfaction than male officers, but this difference was not statistically significant. In contrast, several work environment factors were significantly correlated with intimate relationship satisfaction (Table 4). Perception of mastery (*r* = 0.22, *p* < 0.001), support from friends and relatives (*r* = 0.18, *p* = 0.004), and social climate (*r* = 0.19, *p* = 0.002) showed the strongest positive associations with intimate relationship satisfaction. Other positive correlations included support from coworkers (*r* = 0.15, *p* = 0.02) and fair leadership (*r* = 0.13, *p* = 0.038). Conversely, negative correlations were observed for work centrality (*r* = −0.17, *p* = 0.006), inequality (r = −0.15, *p* = 0.017), and learning demands (*r* = −0.15, *p* = 0.022), indicating that higher levels of these factors were associated with lower intimate relationship satisfaction. Other work environment factors showed no significant associations with intimate relationship satisfaction.

**Table 3:** Bivariate correlation/association between intimate relationship satisfaction and sociodemographic variables.

Variables	Intimate relationship satisfaction (*r* or *t*/*p*)
Gender	1.17/0.242
Female	30.27 ± 12.76
Male	28.05 ± 14.44
Age	0.030/0.638
Children	0.78/0.437
Yes	27.93 ± 13.85
No	29.43 ± 13.66
Job experience	0.082/0.196
Type of work	0.13/0.896
External services	28.85 ± 13.61
Internal services	28.61 ± 14.86
Managerial position	−0.42/0.673
Yes	28.10 ± 14.15
No	28.95 ± 13.94
Shift plan	0.20/0.839
Daytime	29.03 ± 14.10
Two/three-shift	28.66 ± 14

**Table 4:** Bivariate correlations between intimate relationship satisfaction and work environment factors.

Variables	*r*	*p*
Job demands		
Quantitative demands	−0.10	0.121
Decision demands	0.01	0.850
Learning demands	−0.15^*^	0.022
Role expectations		
Role clarity	0.10	0.134
Role conflict	−0.082	0.197
Control at work		
Positive challenges	0.08	0.222
Control of decisions	0.09	0.176
Control of work pace	0.01	0.891
Predictability at work		
Predictability next month	0.06	0.371
Predictability 2 years	0.01	0.915
Preference for challenge	−0.01	0.931
Mastery of work		
Perception of mastery	0.222^**^	<0.001
Social interactions		
Support from supervisor	0.08	0.185
Support from coworkers	0.15^*^	0.020
Support from friends and relatives	0.18^**^	0.004
Bullying	0.07	0.247
Leadership		
Empowering leadership	0.10	0.110
Fair leadership	0.13^*^	0.038
Organizational culture and climate		
Social climate	0.19^**^	0.002
Innovative climate	0.02	0.790
Inequality	−0.15^*^	0.017
Human resources primacy	0.09	0.162
Work centrality	−0.17^**^	0.006
Perception of group work	0.06	0.373
Work motives		
Intrinsic motivation	−0.12	0.070
Extrinsic motivation	0.01	0.855

*r* = Pearson correlation coefficient. *p* = two-tailed significance test.

*p < 0.05, **p < 0.01.

### 3.3 Multiple regression and moderation analysis

A multiple regression analysis was conducted with intimate relationship satisfaction as the dependent variable to examine the combined predictive value of work environment factors on intimate relationship satisfaction. To ensure statistical parsimony and model stability, the selection of predictors was based on both theoretical relevance and significant bivariate associations. Four work environment subscales (perception of mastery, support from friends and relatives, social climate, and work centrality) that demonstrated robust correlations (*p* < 0.001) were included in the model as predictors. Sociodemographic variables (e.g., gender, shift plan, age) were excluded from the final model, as preliminary analyses indicated they did not significantly contribute to the explained variance. This approach was adopted to ensure a sufficient number of participants for each variable in the model, to reduce the risk of overfitting, and to ensure the reliability of the regression coefficients.

The initial regression model was statistically significant, *F*(4, 244) = 8.33, *p* < 0.001, indicating that the combination of the four predictors significantly explained 10.6% of the variance in intimate relationship satisfaction (adjusted *R^2^* = 0.106). Examining the regression coefficients revealed that three of the four predictors significantly contributed to intimate relationship satisfaction. Perception of mastery (*B* = 4.66, *t* = 2.46, *p* = 0.015), social climate (*B* = 2.95, *t* = 2.38, *p* = 0.018), and work centrality (*B* = −2.39, *t* = −3.27, *p* = 0.001) showed significant effects, while support from friends and relatives (*B* = 2.04, *t* = 1.94, *p* = 0.053) approached significance. Analysis of the interactions showed a significant interaction between support from friends and relatives and work centrality (*F* = 4.80, *p* = 0.029). Adding the interaction term (support from friends and relatives * work centrality) improved the model fit, *F*(5, 243) = 7.73, *p* < 0.001 (adjusted *R^2^* = 0.119), explaining 11.9% of the variance (equivalent to a near-medium effect size by Cohen’s (1988) criteria) in intimate relationship satisfaction (Table 5). Social climate also showed a significant positive effect (*B* = 3.11, *p* = 0.012). Work centrality had a significant negative effect (*B* = −2.33, *p* = 0.001). The main effect of support from friends and relatives changed to significant (*B* = 2.20, *p* = 0.036), while perception of mastery was no longer significant (*B* = 3.77, *p* = 0.051), likely because the interaction captured variance previously attributed to perception of mastery. The final moderation model, including the interaction term, is presented in Table 5.

**Table 5:** Multiple regression analysis predicting intimate relationship satisfaction: main effects of workplace factors and the moderating role of work centrality.

Variables	*B*	*SE*	*β*	95% *CI*	*t*	*p*
Constant	28.69	0.83		[27.05, 30.32]	34.53	<0.001
Perception of mastery	3.77	1.92	0.13	[−0.02, 7.56]	1.96	0.051
Social climate	3.11	1.23	0.16	[0.68, 5.54]	2.52	0.012
Support from friends and relatives	2.20	1.05	0.13	[0.14, 4.26]	2.11	0.036
Work centrality	−2.33	0.73	−0.19	[−3.76, −0.90]	−3.22	0.001
Work centrality * Support from friends and relatives	2.08	0.95	0.13	[0.21, 3.95]	2.19	0.029

*N* = 249. Dependent variable = intimate relationship satisfaction. B = unstandardized coefficient. SE = standard error. β = standardized coefficient. CI = confidence interval.

**Figure 1:**
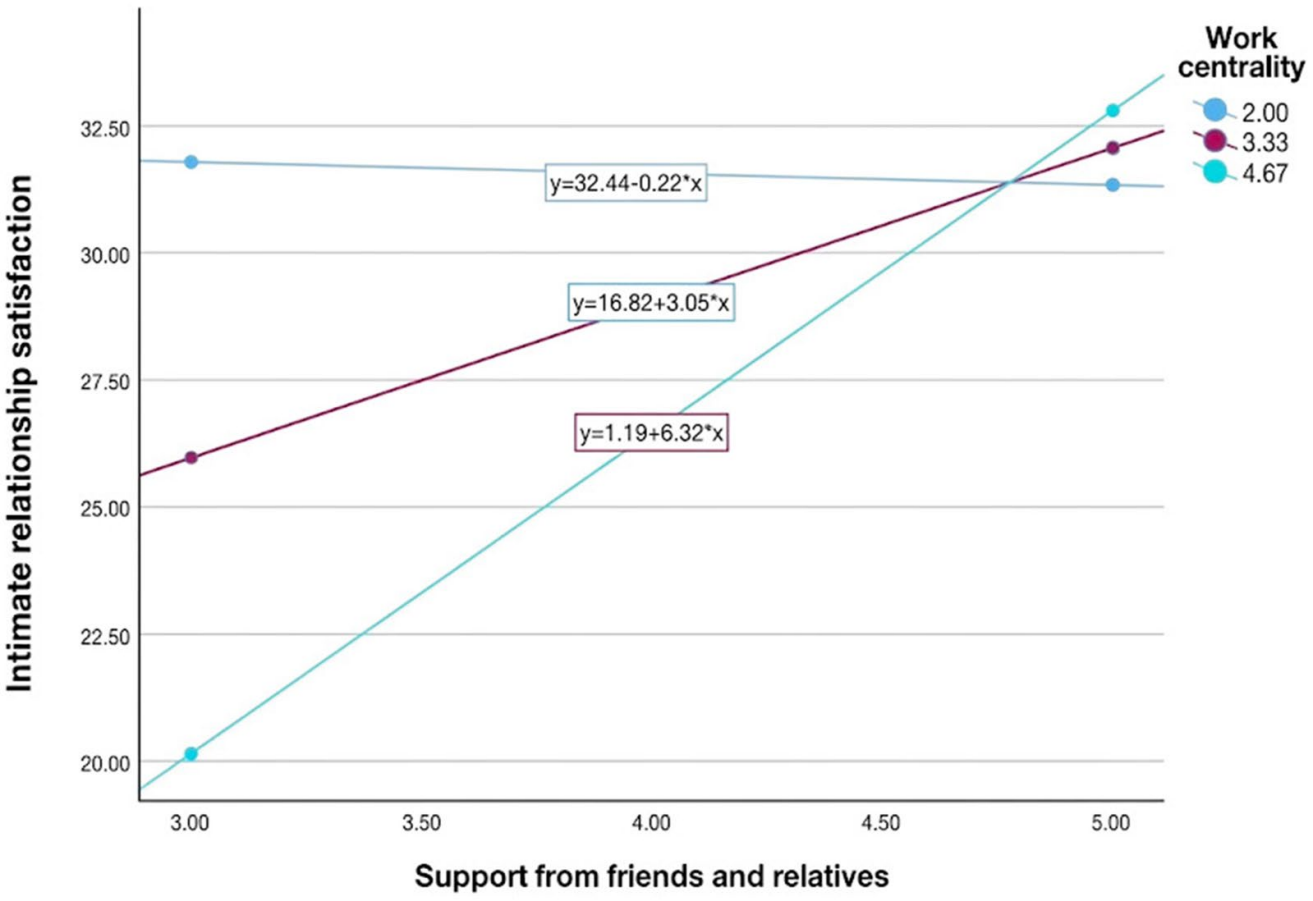
Simple slopes for the interaction effect of work centrality and support from friends and relatives on intimate relationship satisfaction.

To further clarify this interaction, a simple slopes analysis was conducted and is illustrated in Figure 1. The figure shows support from friends and relatives in relation to intimate relationship satisfaction at three levels of work centrality: low (16th percentile; 2.00), medium (50th percentile; 3.33), and high (84th percentile; 4.67). These values were calculated using the pick-a-point approach (see Statistical analysis). Simple slope tests confirmed that the effect of support from friends and relatives on intimate relationship satisfaction was non-significant at low work centrality (*B* = −0.22, *p* = 0.893), but was positive and statistically significant at medium work centrality (*B* = 3.05, *p* = 0.003), and strongest at high work centrality (*B* = 6.32, *p* < 0.001). These findings suggest that support from friends and relatives may be especially beneficial for intimate relationship satisfaction when work centrality is high.

## 4 Discussion

The mean intimate relationship satisfaction score on the EMS scale was 28.75 ± 13.95, indicating a moderate level of satisfaction among the participants (near the study’s specific theoretical midpoint of 33.25). This result reflects a rigorous and conservative estimate of satisfaction, as the formula accounted for a high degree of shared variance (*r^2^* = 0.67) between reported satisfaction and idealistic distortion (*r* = 0.82). This suggests that while police officers in this context may tend to present their relationships in an idealized light, the adjusted EMS score successfully provided a grounded, moderate level of satisfaction. The relatively large standard deviation and the spread across quartiles indicate meaningful variability in intimate relationship satisfaction, suggesting that officers differ substantially in their perceived relationship quality. Since the scoring and categorization were based on an internal percentile approach rather than external norms, the results provide a localized benchmark. This approach offers a more accurate reflection of this population than external normative data.

Sociodemographic variables (gender, age, education, and having children) and work-related characteristics (type of police work, job experience, and shift schedules) were not significantly associated with intimate relationship satisfaction, suggesting that these factors may play a limited role for intimate relationship satisfaction in this sample. Research on the role of sociodemographic factors has reported mixed results. For instance, some studies on gender differences in intimate relationship satisfaction have found that men report higher levels of satisfaction than women (Boerner et al., 2014; Rostami et al., 2014), whereas a meta-analysis identified a slight gender difference in the general population, with women reporting lower satisfaction than men (Jackson et al., 2014). Further research with larger and more diverse samples of police officers is needed to elaborate on the role of these factors in this context.

In the bivariate correlations, perception of mastery showed a significant positive association with intimate relationship satisfaction. This is consistent with the positive spillover hypothesis (Hanson et al., 2006), which suggests that success and satisfaction in one life domain can enhance well-being in another. Experiences of mastery and competence foster generalized confidence and self-regulation, which extend across life domains (Bandura, 1997). Our results suggest that when police officers feel capable of handling work challenges, they may be less likely to experience stress or exhaustion that spills over into family life, thereby reducing work–family conflict and enhancing intimate relationship satisfaction (Greenhaus and Beutell, 1985; Schieman and Narisada, 2014). From a conservation of resources perspective (Hobfoll, 1989; Hobfoll et al., 2018), mastery at work serves as a personal resource that may buffer against the resource loss caused by high work pressure. Consistent with the crossover effect, a sense of mastery may foster feelings of accomplishment that potentially extend to the family domain. However, in the final multiple regression model including the interaction between support from friends and relatives and work centrality, the effect of mastery became marginally non-significant (*p* = 0.051). This finding suggests that the relationship between mastery and intimate relationship satisfaction may be partially accounted for by other variables in the model, particularly work centrality and social support. This underscores the importance of examining work–family relationships within multifactorial models rather than relying on single predictors.

The findings indicate that a positive social climate at work, characterized by support, encouragement, safety, trust, and pleasantness, is associated with greater satisfaction in intimate relationships. This relation may be particularly important in police work, where the organizational climate is often characterized in research as competitive and rigid, and professional competence and value in policing are demonstrated through competition, toughness, and emotional restraint (Berdahl et al., 2018). Such a social climate can heighten stress and pressure, depleting police officers’ psychological resources and thus negatively affecting their well-being and ability to maintain work–family balance and intimate relationships (Munsch et al., 2018; Buhrig, 2023). From the perspective of the conservation of resources theory (Hobfoll, 1989), a positive work climate can serve as a job resource that may help officers conserve and replenish their personal resources depleted by the high demands of police work. This resource empowers them to cope with work-related pressure and burnout (Wolter et al., 2019; Zeng et al., 2020; Johnson and Shamroukh, 2024; Kiptulon et al., 2024). Police officers who possess more resources are theoretically less vulnerable to losing them and more likely to gain additional resources (Hobfoll et al., 2018), which may help as protective factors in overcoming work demands and managing work–family conflicts (Antunes et al., 2024). Without such resources, the negative spillover of work–family conflict can undermine intimate-relationship satisfaction (Roberts and Levenson, 2001; Tuttle et al., 2018). Our results indicated a significant negative relationship between work centrality and intimate relationship satisfaction. In the multiple regression analysis, work centrality was also the variable most strongly and negatively associated with satisfaction in our model. Work centrality reflects the importance and position of work in an individual’s life, the extent to which they allocate their personal resources to work compared to other areas of life such as their family (Paullay et al., 1994; Bagger and Li, 2012; Moura and Silva, 2019). The culture of police work, characterized by a strong work culture that emphasizes loyalty, solidarity, and commitment to police work and the police family, may make work centrality more pronounced (Loftus, 2010; Reiner, 2010; du Plessis et al., 2021). This organizational culture can encourage strong identification with the job and work centrality, positioning the police role as the main priority and central interest of officers’ lives (Filstad, 2022). Higher work centrality is associated with longer working hours and greater work engagement (Bal and Kooij, 2011; Kostek, 2012), which may lead to further investment of individual resources in work, resulting in resource loss in family life (Carr et al., 2008; Hobfoll, 2011; Basinska and Dåderman, 2019). Research has shown that higher work centrality and work engagement are associated with greater work stress and work–family conflicts in police officers (Frone et al., 1992; Liu et al., 2019). According to the conservation of resources theory and work–family conflict theory, if the depletion of personal resources caused by high work centrality cannot be replenished or recovered, it may intensify family conflict by increasing both time-based and emotion-based conflicts. While other research has shown that work centrality is associated with several positive outcomes, such as organizational commitment, job satisfaction, job performance, and lower turnover (Carr et al., 2008; Tziner et al., 2014; Tan, 2016), our findings suggest that in the context of policing, high work centrality may be associated with an imbalanced life orientation that challenges officers’ family life and intimate relationship satisfaction.

A particularly novel finding of this study is the significant interaction between work centrality and support from friends and relatives, indicating that work centrality moderates the relationship between support and intimate relationship satisfaction. While there is a positive bivariate association between support from friends and relatives and intimate relationship satisfaction, the interaction effect in the multiple regression analysis shows a conditional relationship in which the predictive value of social support and intimate relationship satisfaction significantly increases from negligible at low work centrality to significant at high work centrality. This finding indicates that social support is not uniformly beneficial but becomes increasingly important as work becomes more central to police officers. This conditional effect can be explained by the conservation of resources theory—that social support provides individuals with access to valuable external resources that complement, recover, or enrich their internal personal resources (Hobfoll et al., 1990). Theoretically, officers with high work centrality allocate more personal resources to the work domain, which could lead to resource scarcity in the marital domain. In this situation, social support from outside of work provides an external resource and compensates for the attention and emotional energy dedicated toward work, resulting in the mitigation of work–family conflict and higher intimate relationship satisfaction (Michel et al., 2011; Kelley et al., 2021; Neculman et al., 2025). Conversely, when work centrality is low, the investment of resources appears to be already directed toward the family rather than work; the effect of external support thus becomes negligible. This pattern underscores the conditional role of social support, indicating that its effect depends on how central work is in an officer’s life and how they allocate their personal resources.

The present study has several limitations. The current study focused on a specific sample of police officers recruited from certain geographic regions in Sweden. Although the Swedish Police Authority is a national organization with standardized training and protocols, which supports the representativeness of the findings within the Swedish context, this geographic focus may restrict generalizability to different policing cultures or broader populations. More research with a larger, more diverse sample would help validate these results. Moreover, the study targeted officers in committed relationships to satisfy the requirements of the EMS scale. This excludes single officers and those in less committed relationships, potentially introducing a selection bias toward more stable domestic situations. Furthermore, although the EMS scale is well known, it was originally validated in the United States and may not fully capture intimate relationship satisfaction within a European cultural context. Furthermore, in the absence of European normative data, a sample-specific constant was calculated. While these adaptations were necessary to ensure contextual relevance, they limit the direct comparability of our findings with existing US-based normative data and other studies utilizing the original marital version of the scale. Another limitation of this study was the reliance on individual self-report data from one of the partners. Incorporating dyadic data from police officers and their partners could provide a more accurate and nuanced picture of how work-related factors relate to family relationships than the perspective of one individual alone. Furthermore, although quantitative methods can provide valuable correlational insights, they may overlook the more nuanced aspects of police officers’ family life and the various factors influencing it. In addition, the exclusion of the scale on the interaction between work and private life from the questionnaire limits further exploration of the direct association between work–family interaction and intimate relationship outcomes. Future research should prioritize this scale to enhance our understanding of work–family dynamics. The integration of qualitative approaches could deepen the understanding of the complicated relationship between work life and intimate relationships. Finally, the study was conducted within a specific cultural context, Sweden, characterized by high gender equality, institutionalized work–life balance norms, and a strong social welfare system. These cultural conditions may shape both the experience of work–family conflict and the subjective assessment of intimate relationship satisfaction in ways that are not captured by the study variables. Therefore, findings may not generalize to policing contexts with different cultural values regarding work, family, and gender roles. Future research would benefit from explicitly incorporating cultural dimensions to better understand how these processes may vary across different societal contexts.

### 4.1 Implications

The results of this study contribute to the literature on work life and intimate relationships within the context of the police work environment. The findings have practical implications for both police organizations and police officers, especially in a police context such as that in Sweden, where recent organizational reforms and increased violent crime have intensified organizational and individual challenges within the force. The most theoretically significant finding of this study is the significant interaction between work centrality and support from friends and relatives. This moderation effect extends the conservation of resources theory by specifying a boundary condition: external social support does not uniformly buffer work–family conflict, but functions as a compensatory resource specifically when internal resources have been depleted by high work centrality. This finding contributes theoretically by identifying when and for whom external support matters most, moving beyond main-effect predictions toward a more conditional and dynamic understanding of resource compensation in the work–family interface.

While the model explained approximately 12% of the variance in intimate relationship satisfaction, this should be interpreted in light of the inherently complex and subjective nature of the outcome variable. Intimate relationship satisfaction is shaped by numerous factors, including personality traits, partner dynamics, communication patterns, and broader life circumstances, which were not included in this study. The finding that work environment factors alone account for nearly 12% of the variance is therefore theoretically meaningful and practically significant, particularly within the context of organizational intervention. Importantly, the modest R^2^ does not diminish the significance of the observed individual associations; rather, it highlights that this study captures an important piece of a larger, multifaceted picture. Future research integrating work-related, interpersonal, and cultural variables in a single model would provide a more comprehensive account of intimate relationship satisfaction among police officers.

The negative association between work centrality and intimate relationship satisfaction highlights the importance of maintaining a delicate balance between being a committed and dedicated police officer (police identity) and fulfilling other roles and identities outside of work. Furthermore, the protective role of external social support when work centrality levels are high suggests that social networks outside the policing context are important. Organizations could facilitate this through policies that protect off-duty time, normalize work disengagement, and provide resources to help officers and their families maintain connections outside of work. Given the significance of the workplace social climate for police officers’ satisfaction in intimate relationships, organizations play a crucial role in fostering a more trusting, collaborative, supportive, and encouraging work environment for police officers. Such changes could enhance both the family life and the work performance of police officers. Family involvement in officer support programs may also serve as a valuable additional resource, mitigating the negative associations between work demands and officers’ personal relationships.

### 4.2 Conclusion

This study offers valuable insights into intimate relationship satisfaction among Swedish police officers and how different aspects of the police work environment are related to this satisfaction. In general, participants reported moderate satisfaction levels in their intimate partner relationships. High centrality of work is negatively associated with intimate relationship satisfaction, where a positive social climate at work and social support from friends and relatives appear to serve as important protective factors. Interestingly, the interaction between work centrality and external support suggests that when one’s job becomes a core part of one’s life, having a strong social network outside work plays a crucial role in maintaining healthy intimate relationships. Work centrality among police officers can reflect a deep commitment to their role, a strong professional identity, and a sense of purpose, all of which serve as resources of personal fulfillment. However, the findings of this study suggest that when work becomes so central that it can overshadow the intimate relationship, it can act as a risk factor for relationship satisfaction. Thus, police organizations should approach work centrality as a double-edged sword for the professional and personal lives of police officers, which can benefit organizational functioning and individual professional growth, but is simultaneously linked to challenges in personal intimate relationships. The study suggests that both organizational policies and counseling initiatives could focus more on strengthening external support systems and involving families in supportive ways. The potential negative impact of high work centrality on marriage can be mitigated when both individuals and the police organization actively foster and maintain robust external and organizational support systems. The findings highlight the importance of adopting a holistic approach that combines organizational change, family-oriented support, and individual-level strategies to enhance sustainable work–family balance in policing.

## Data Availability

The data analyzed in this study is subject to the following licenses/restrictions: according to the target group of the study (police officers), dataset cannot be publicly available. Requests to access these datasets should be directed to arian.rostami@umu.se.
